# Polycomb Repressive Complex 2 attenuates the very high expression of the *Arabidopsis* gene *NRT2.1*

**DOI:** 10.1038/s41598-018-26349-w

**Published:** 2018-05-21

**Authors:** Fanny Bellegarde, Léo Herbert, David Séré, Erwann Caillieux, Jossia Boucherez, Cécile Fizames, François Roudier, Alain Gojon, Antoine Martin

**Affiliations:** 1 BPMP, CNRS, INRA, SupAgro, Univ. Montpellier, Montpellier, France; 2 Institut de Biologie de l’Ecole Normale Supérieure, CNRS UMR8197, INSERM U1024, ENS, 46 rue d’Ulm, 75005 Paris, France; 3 Laboratoire Reproduction et Développement des Plantes, Univ Lyon, ENS de Lyon, UCB Lyon 1, CNRS, INRA, F-69342 Lyon, France

## Abstract

PRC2 is a major regulator of gene expression in eukaryotes. It catalyzes the repressive chromatin mark H3K27me3, which leads to very low expression of target genes. *NRT2.1*, which encodes a key root nitrate transporter in *Arabidopsis*, is targeted by H3K27me3, but the function of PRC2 on *NRT2.1* remains unclear. Here, we demonstrate that PRC2 directly targets and down-regulates *NRT2.1*, but in a context of very high transcription, in nutritional conditions where this gene is one of the most highly expressed genes in the transcriptome. Indeed, the mutation of *CLF*, which encodes a PRC2 subunit, leads to a loss of H3K27me3 at *NRT2.1* and results, exclusively under permissive conditions for *NRT2.1*, in a further increase in *NRT2.1* expression, and specifically in tissues where *NRT2.1* is normally expressed. Therefore, our data indicates that PRC2 tempers the hyperactivity of *NRT2.1* in a context of very strong transcription. This reveals an original function of PRC2 in the control of the expression of a highly expressed gene in *Arabidopsis*.

## Introduction

Polycomb Repressive Complex 2 (PRC2) is a major and conserved regulatory complex of gene expression in eukaryotes. PRC2 is essential for growth and development and in both plants and animals loss-of-function of PRC2 subunits leads to serious phenotypic defects^1,2^. PRC2-mediated regulation of gene expression relies on the modification of chromatin state, by catalyzing the tri-methylation of Lys 27 of histone H3 (H3K27me3). Point mutation in H3K27 leads to similar phenotypes to those of PRC2 mutants, demonstrating that H3K27me3 is the main effector of PRC2-mediated regulation^3^. In *Arabidopsis*, CURLY LEAF (CLF) and SWINGER (SWN) are two different PRC2 enzymatic subunits that tri-methylate H3K27 in vegetative tissues^2^. CLF and SWN are thought to have overlapping functions, but the predominant contribution of CLF to H3K27me3 enrichment, as well as the more severe phenotype of *clf* mutant plants compared to that of *swn* mutant plants, suggest that CLF is the major H3K27 tri-methyltransferase during *Arabidopsis* vegetative development^4,5^. Although the molecular mechanisms by which PRC2 and H3K27me3 mediate transcriptional regulation are not fully understood, a large number of epigenomic analyses have demonstrated that H3K27me3 and PRC2 members are associated with strong repression of gene expression^5–9^. In the *Arabidopsis* genome, 20–25% of genes are marked by H3K27me3 and globally display low or very low expression^6,7^, and mutations in CLF lead to up-regulation of several hundred H3K27me3-associated genes^5,10^.

Many genes controlled by PRC2-mediated H3K27me3 levels in *Arabidopsis* correspond to genes involved in the regulation of development, and in particular transcription factors^5,6,8,9,11^. One of the best described examples corresponds to the repression of the *FLOWERING LOCUS C* (*FLC*) gene. *FLC* repression depends on H3K27me3 enrichment, and further experiments have demonstrated that *FLC* exists in bistable on/off expression states whether it is marked or not by H3K27me3, suggesting that H3K27me3 is a major molecular determinant of strong gene repression^12,13^. On the other hand, H3K27me3 levels have been also proposed to quantitatively regulate gene expression. This has been notably illustrated by the effect of mutations for *PRC2* subunits in the control of the rate of induction of the *VERNALIZATION INSENSITIVE 3 (VIN3)* gene in response to cold treatment^14^.

PRC2 target loci also often correspond to genes showing tissue-specific expression. Such genes are heavily marked with H3K27me3 in the tissues where they are silent, and at the reverse largely depleted in H3K27me3 enrichment in tissues where they are normally expressed^11,15^. Accordingly, numerous studies have described in *Arabidopsis* that loss of PRC2-mediated regulation leads to an aberrant expansion of the expression territory of tissue-specific genes^10,16–19^. Altogether, the observations listed above led to the conclusion that PRC2 and associated H3K27me3 enrichment are strong negative transcriptional regulators ensuring the correct spatio-temporal pattern of expression of developmental genes. Nevertheless, decrease in PRC2-mediated H3K27me3 levels on target genes is not systematically associated with increase in gene expression or modifications of tissue-specific expression pattern^20–22^.

In *Arabidopsis*, *NITRATE TRANSPORTER 2.1 (NRT2.1)* encodes a key high-affinity root nitrate (NO_3_^−^) transporter, crucial for root uptake of NO_3_^−^ and thus for nitrogen (N) nutrition of the plant^23,24^. Accordingly, *nrt2.1* mutants show a dramatic reduction of growth under low and limiting NO_3_^−^ availability^23,25,26^. In agreement with its major physiological role, the *NRT2.1* gene is strongly regulated at the transcriptional level by environmental factors affecting root NO_3_^−^ uptake^27^. In particular, *NRT2.1* is very differentially expressed depending on the level of N supply, with very low expression under N-rich media, and exceptionally high expression under low and limiting NO_3_^−^ availability^28^. In addition, *NRT2.1* displays a very strict tissue-specific transcriptional profile, with expression confined to the outer layers of the root tissues^29,30^. It has recently been observed that *NRT2.1* is marked by H3K27me3^6,9^, indicating that PRC2 activity could be a potential determinant of the repression of *NRT2.1* gene expression under N-rich condition^31^. In contrast to the regulation of genes involved in cell differentiation and plant development, the role of PRC2 in the regulation of environmentally-responsive and nutrition-related genes like *NRT2.1* remains to be fully investigated.

To address this question, we investigated in detail the role of H3K27me3 and PRC2 in the regulation of *NRT2.1* expression under both strongly repressive (high N supply) or highly inductive (low NO_3_^−^ availability) conditions. We unexpectedly found that PRC2 downregulates *NRT2.1* expression only in a context of very strong transcription, and specifically in tissues where *NRT2.1* is highly expressed. We observed that a loss of H3K27me3 under conditions of very high expression results in a further increase in *NRT2.1* promoter activity. We thus reveal here an original role for PRC2 in modulating the transcriptional level of *NRT2.1* specifically under conditions where it is one of the most highly expressed genes in *Arabidopsis* roots.

## Results

### PRC2 directly regulates *NRT2.1* in the context of very strong expression

**Figure 1:**
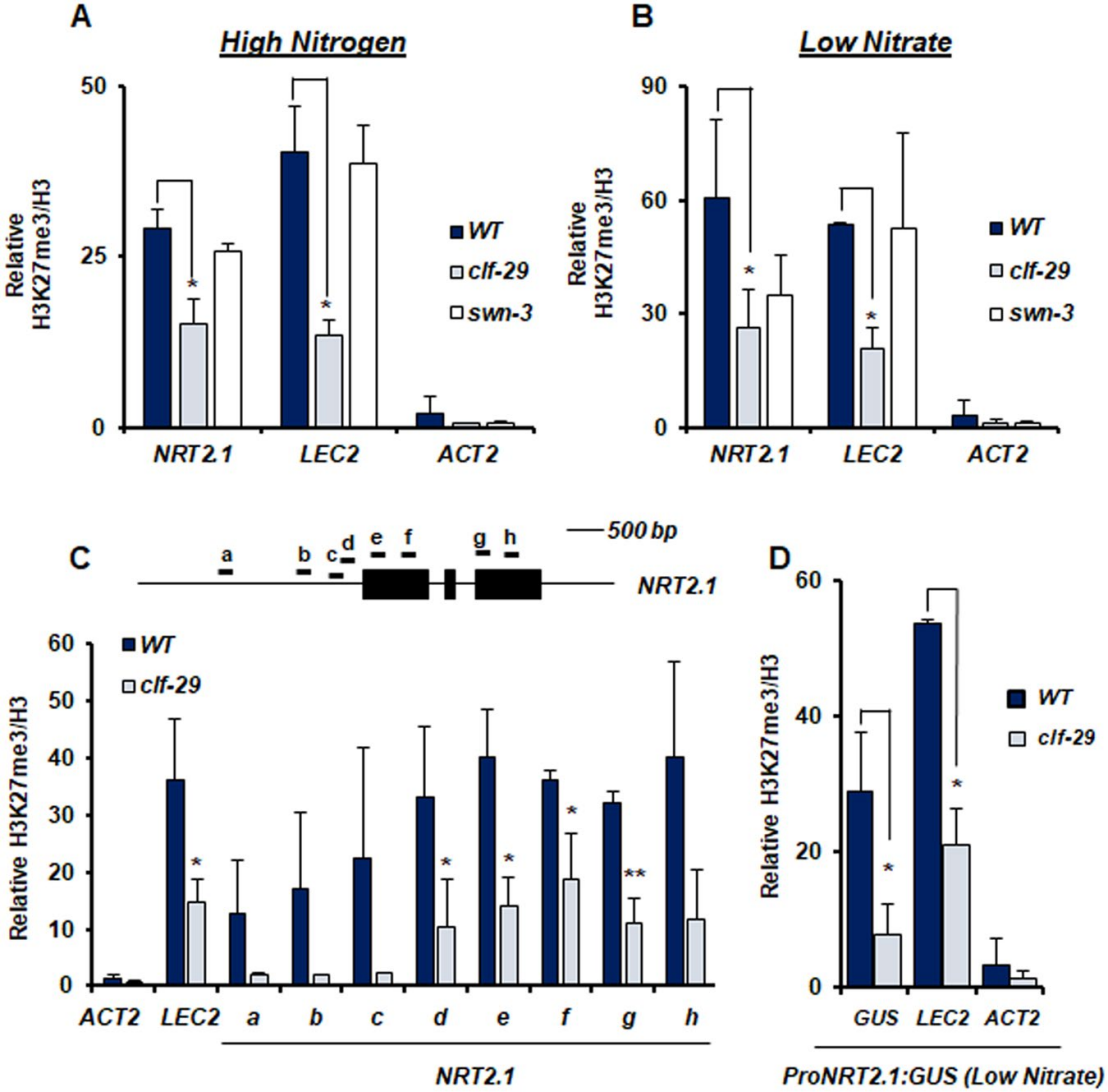
CLF controls H3K27me3 enrichment at the *NRT2.1* locus under both repressive and active conditions for expression. ChIP analysis of H3K27me3 in *WT*, *clf-29*, and *swn-3* roots of 7 days-old plants grown under (**A**) high nitrogen (10 mM NH_4_NO_3_) or (**B**) low nitrate (0.3 mM NO_3_^−^) conditions. *LEC2* and *ACT2* served as positive or negative control for H3K27me3, respectively. Positions of primers used in qRT-PCR are available in Fig. S1. (**C**) ChIP analysis of H3K27me3 in *WT* and *clf-29* covering the *NRT2.1* locus. (**D**) ChIP analysis of H3K27me3 at the *ProNRT2.1:GUS* locus in *WT* and *clf-29* roots of 7 days-old plants grown under low nitrate (0.3 mM NO_3_^−^) condition. Quantification by qRT-PCR is shown as the percentage of H3. Error bars represent standard errors of the mean based on 3 biological replicates. Statistical significance was computed using a two-tailed Student’s t-test. Significance cutoff: *p < 0.05, **p < 0.01, ***p < 0.001.

**Figure 2:**
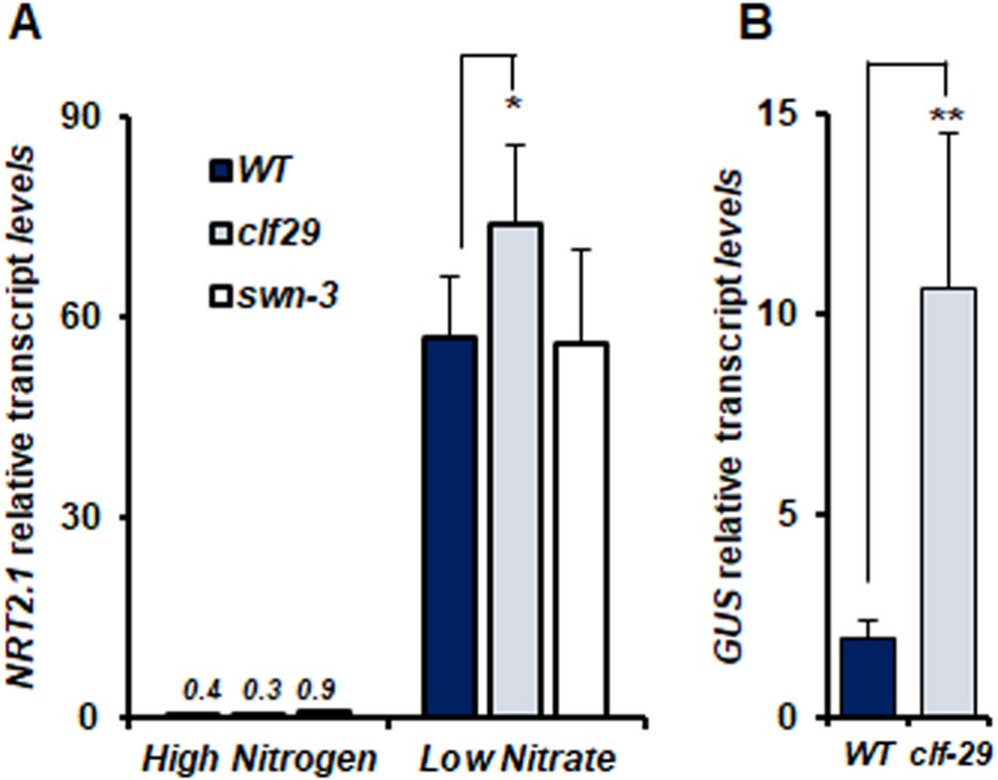
Reduction of H3K27me3 in *clf-29* increases the expression of *NRT2.1* in a context of very high expression. (**A**) Relative expression of *NRT2.1* by qRT-PCR in roots of 7-days old of *WT*, *clf-29*, and *swn-3* plants grown under high nitrogen (10 mM NH_4_NO_3_) or low nitrate (0.3 mM NO_3_^−^) conditions. Quantification by qRT-PCR is shown as the percentage of *ACT2* transcript levels. (**B**) Relative *GUS* expression by qRT-PCR in roots of 7-days old *ProNRT2.1:GUS WT* and *clf-29* plants grown under low nitrate (0.3 mM NO_3_^−^) condition. Quantification by qRT-PCR is shown as the percentage of *ACT2* transcript levels. Error bars represent standard errors of the mean based on 3 biological replicates. Statistical significance was computed using a two-tailed Student’s t-test. Significance cutoff: *p < 0.05, **p < 0.01, ***p < 0.001.

*NRT2.1* is differentially expressed depending on the level of N supply, with very low expression under N-rich media, and very high expression under low and limiting NO_3_^−^ availability^28^. To investigate the role of PRC2 in *NRT2.1* regulation, we measured H3K27me3 enrichment at the *NRT2.1* locus in WT and mutant lines for CLF and SWN, under highly contrasted conditions for expression, and compared it with an actively transcribed gene (*ACTIN2*, *ACT2*) or a known PRC2 target gene (*LEAFY COTYLEDON 2, LEC2*). Under N-rich repressive conditions, H3K27me3 enrichment at the *NRT2.1* locus was indeed elevated in the roots of a WT line, and significantly reduced in *clf-29* mutant but not in *swn-3* mutant (Figs 1A and S1 for information about primers position). Under a NO_3_^−^ limiting environment, which corresponds to the most favorable condition for *NRT2.1* expression^29^, we surprisingly also observed a strong H3K27me3 enrichment at the *NRT2.1* locus, similar to those observed for typical PRC2-controlled genes such as *LEC2* (Fig. 1B). This was completely unexpected as, strikingly, *NRT2.1* is ranked among the 3 most highly expressed genes in the whole *Arabidopsis* root transcriptome obtained under exactly the same NO_3_^−^ limiting condition (Table S1). Under NO_3_^−^ limitation, H3K27me3 levels at the *NRT2.1* locus were also significantly diminished in *clf-29*, and not in *swn-3* (Fig. 1B), revealing that CLF is the main methyltransferase operating at the *NRT2.1* locus. In order to have a more complete view of the effect of *clf* mutation at the *NRT2.1* locus, we screened the whole locus for H3K27me3 enrichment in WT and *clf-29* lines. In agreement with published epigenomic dataset, a high H3K27me3 enrichment was observed in the *NRT2.1* promoter and in the 5’ part of the gene, and was maintained throughout the whole *NRT2.1* gene body (Figs 1C and S2). Reduction of H3K27me3 enrichment at the *NRT2.1* locus in *clf-29* was found throughout all the locus, but the extent of the reduction was maximal at the promoter region (Fig. 1C). When we measured *NRT2.1* transcript levels in WT, *clf-29* and *swn-3* lines, we observed that decrease in H3K27me3 levels under N-rich repressive conditions did not lead to induction of *NRT2.1* expression, which is, under this condition, still close to zero in mutant lines for PRC2 components (Fig. 2A). Surprisingly, and unlike under repressive N-rich condition, we observed that the reduction of H3K27me3 enrichment in *clf-29* mutant under NO_3_^−^ limitation led to significantly higher *NRT2.1* transcripts level than in the WT line (Fig. 2A). This unexpectedly suggests that PRC2, and in particular CLF, regulates *NRT2.1* in a context of very strong expression. Since we observed that the effect of *clf* mutation on H3K27me3 enrichment at the *NRT2.1* locus was maximal at the promoter region, we crossed the *clf-29* mutant line with the reporter construct *ProNRT2.1:GUS*^29^*. ProNRT2.1:GUS* reporter gene has been previously characterized, and faithfully transposes the transcriptional regulations targeted to *NRT2.1*, including N-responsiveness and tissue-specificity expression^29^. We compared, specifically under NO_3_^−^ limitation, changes in transcript levels and H3K27me3 enrichment at the *ProNRT2.1:GUS* locus in WT and *clf-29* plants. Under NO_3_^−^ limitation, we observed in the *clf-29* mutant a strong increase in *GUS* transcripts level (Fig. 2B). Strikingly, the induction of *GUS* expression in *clf-29* was higher than the one of *NRT2.1*, again suggesting that the regulation mediated by CLF is mainly directed to the promoter activity. In agreement with these observations, we actually found a strong H3K27me3 enrichment at the *GUS* locus in a WT line, and a reduction of this enrichment in the *clf-29* mutant (Fig. 1D). This means that the *NRT2.1* promoter is able to instruct H3K27me3 enrichment to downstream sequences, and that the *ProNRT2.1:GUS* follows the same behavior as *NRT2.1* in response to *clf* mutation. In order to further confirm our observations, we crossed an independent *ProNRT2.1:LUC* transcriptional reporter line^28^ with the *clf-29* mutant. We observed an increase in *LUC* transcripts level in the *clf-29* mutant as compared to the WT background, confirming our observations made on *NRT2.1* and *ProNRT2.1:GUS* (Fig. S3).

To support the observation that CLF regulates *NRT2.1* in a context of very high expression, we checked the presence of CLF at the *NRT2.1* locus in this condition. We therefore performed ChIP using a *ProCLF:CFP:CLF;clf-29* line^17^ to test whether *NRT2.1* is bound by CLF. In comparison to negative and positive controls, we found that CLF indeed associates with the *NRT2.1* locus (Fig. S4). As mutations in CLF lead to up-regulation of several hundred genes, we also checked that the expression of transcriptional regulators of *NRT2.1* under NO_3_^−^ limitation was not perturbed in *clf-29*. We therefore measured, in WT and *clf-29* lines, transcript levels for the main transcriptional regulators of *NRT2.1* that have been previously identified^32^. None of the *NRT2.1* transcriptional regulator that we tested shows a significant de-regulation in *clf-29* (Fig. S5), strongly reinforcing the idea of a direct action of CLF-PRC2 in the regulation of *NRT2*.1 under highly permissive condition for expression.

**Figure 3:**
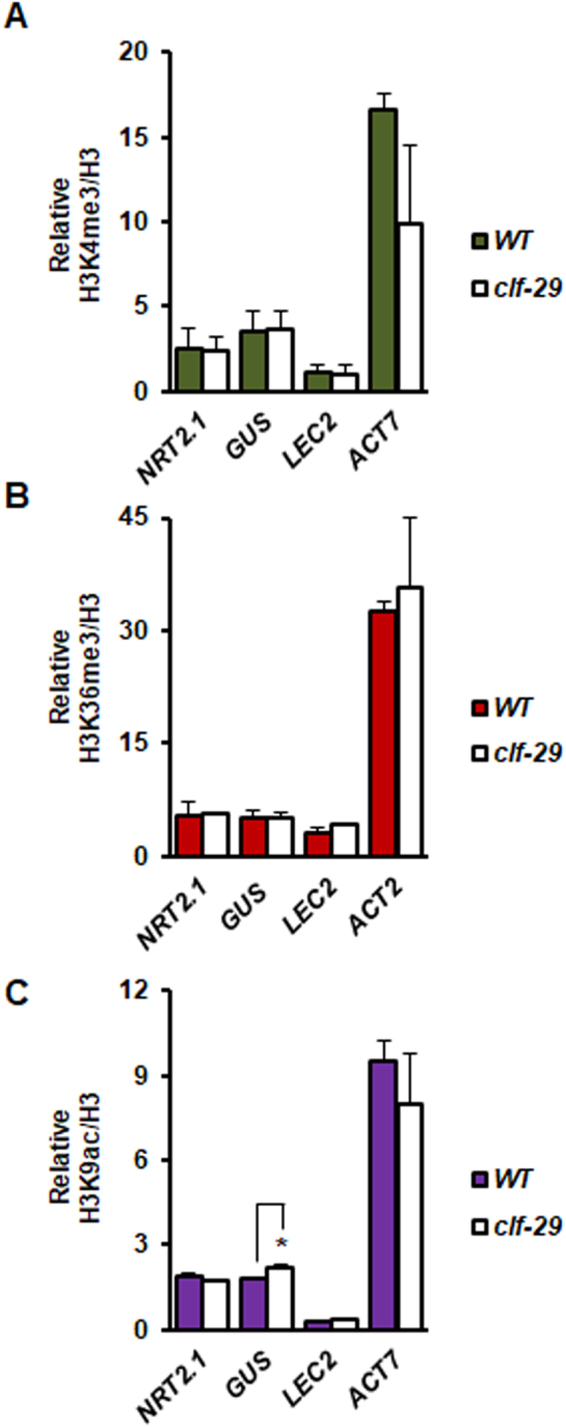
Reduction of H3K27me3 in *clf-29* in the context of active transcription does not lead to an increase in H3K4me3, H3K36me3 or H3K9ac at the *NRT2.1* locus. ChIP analysis of (**A**) H3K4me3, (**B**) H3K36me3, (**C**) H3K9ac in *WT* and *clf-29* roots of 7 days-old plants grown under low nitrate (0.3 mM NO_3_^−^) condition. Quantification by qRT-PCR is shown as the percentage of H3. *ACT7* served as positive for H3K4me3 and H3K9ac, *ACT2* served as positive for H3K36me3, *LEC2* served as negative control for H3K4me3, H3K36me3 and H3K9ac. Error bars represent standard errors of the mean based on at least 3 biological replicates. Statistical significance was computed using a two-tailed Student’s t-test. Significance cutoff: *p < 0.05, **p < 0.01, ***p < 0.001.

To further analyze the chromatin-based regulation of *NRT2.1* by CLF under inductive conditions, we analyzed specifically under NO_3_^−^ limitation the pattern of chromatin marks associated with transcriptional activation. In the WT, *NRT2.1* was surprisingly weakly enriched in H3K4me3, H3K36me3 and H3K9ac (Fig. 3A,B and C), in spite of very high expression levels. In the *clf-29* mutant, reduction of H3K27me3 level and higher transcripts level were not associated with an increase in any of the chromatin marks associated with transcriptional activation (Fig. 3A,B and C). We observed for active chromatin marks at the *ProNRT2.1:GUS* the same profile as the one observed at the *NRT2.1* locus, except a slight increase in H3K9ac enrichment in *clf-29*, in agreement with a higher induction of expression for *ProNRT2.1:GUS* than for *NRT2.1* (Fig. 3C). This suggests that reduction of H3K27me3 could be by itself the cause of the overexpression of *NRT2.1*. All together, these results led us to conclude that the absence of functional PRC2, and subsequent reduction in H3K27me3 levels, consequently increase *NRT2.1* expression, exclusively under highly permissive NO_3_^−^ limiting condition.

### PRC2 modulates the expression of *NRT2.1* specifically in *NRT2.1*-expressing tissues

**Figure 4:**
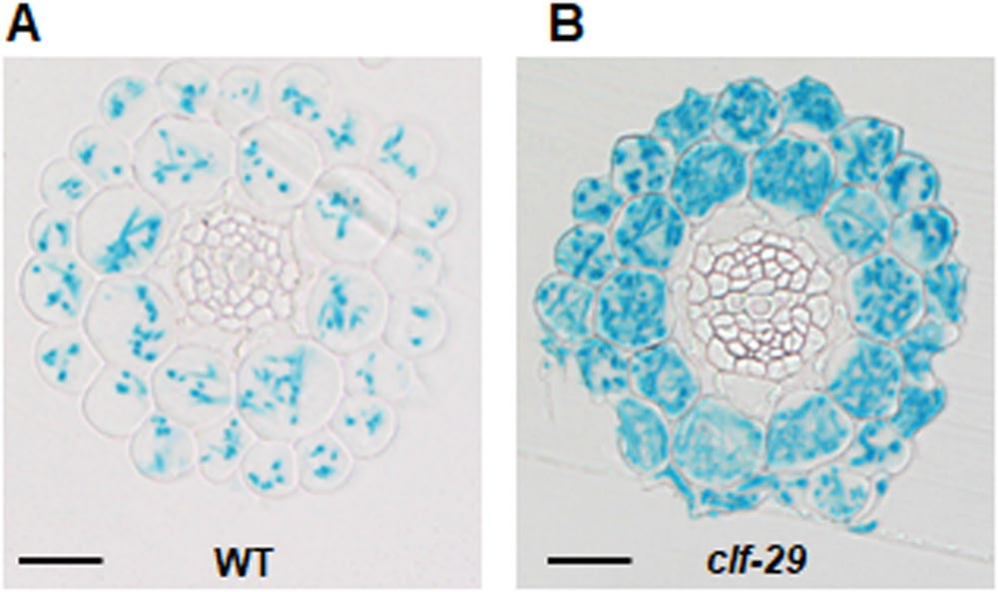
CLF and H3K27me3 control *NRT2.1* expression in *NRT2.1*-expressing root tissues. Histochemical localization of GUS expression on root transversal sections of 7 days-old *Arabidopsis WT* (**A**) and *clf*-*29* (**B**) lines containing *ProNRT21:GUS*, and grown under low nitrate (0.3 mM NO_3_^−^) condition. Bar = 25 µm.

PRC2 and associated H3K27me3 enrichment are strong negative transcriptional regulators, which also ensure the correct spatio-temporal expression pattern of target genes. We therefore addressed the question whether down-regulation of *NRT2.1* by CLF under highly inductive conditions corresponds to transcriptional repression in tissues where *NRT2.1* is not expressed, or to modulation of expression in tissues where *NRT2.1* is strongly expressed. Since *ProNRT2.1:GUS* faithfully transposes chromatin-based regulation of *NRT2.1* by CLF, we first performed transversal root sections using the WT or *clf-29* lines containing the *ProNRT2.1:GUS* reporter grown under highly inductive NO_3_^−^ limiting condition. In the WT, as previously described, we observed that *NRT2.1* expression is confined, in a very strict manner, to the outer tissues of the root (cortex and epidermis) (Fig. 4A). Strikingly, *NRT2.1* expression in *clf-29* was similarly confined in cortex and epidermis, showing that tissue-specific expression of *NRT2.1* is maintained in spite of a decrease in H3K27me3 enrichment. On the other hand, we observed in *clf-29* specifically and homogeneously in every cortex or epidermis cell a strong increase in GUS staining, reflecting the overexpression *ProNRT2.1* activity specifically in these tissues (Fig. 4B). Altogether, our results demonstrate that the level of H3K27me3, which has been fully characterized as a repressive chromatin mark associated with strongly repressed genes, directly modulates the expression of *NRT2.1*, one of the most highly expressed genes in the transcriptome under limiting NO_3_^−^ availability.

### Genes with very strong expression targeted by H3K27me3 are principally involved in response to stimulus, metabolism, and nutrition

**Figure 5:**
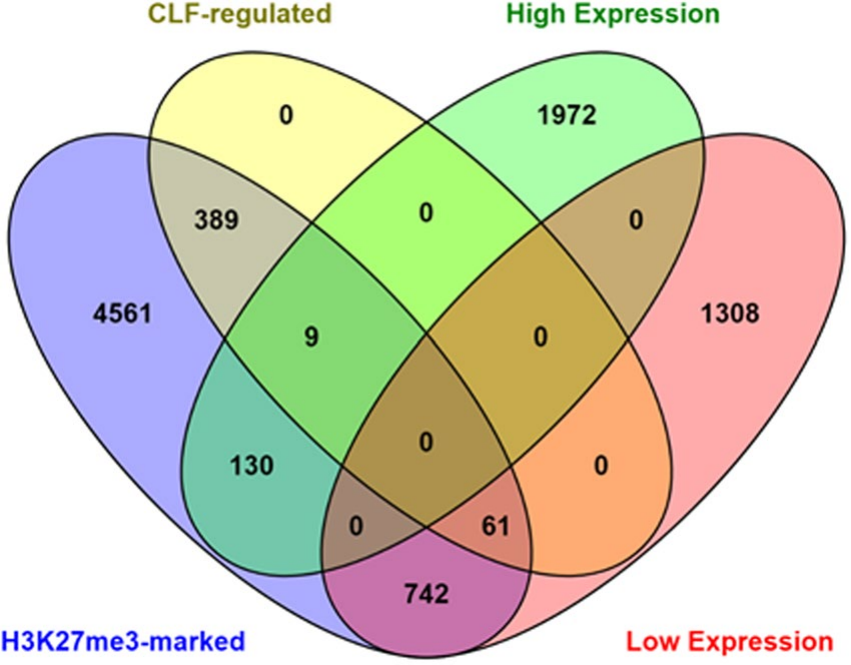
Comparison of genes with very low or high expression showing H3K27me3 enrichment and regulation by CLF. Venn diagram representing a comparison of the proportion of genes marked by H3K27me3 and regulated by CLF among the very low expressed or very highly expressed genes. Very low expressed genes correspond to the 10 percent of genes the most poorly expressed in the transcriptome; very highly expressed genes correspond to the 10 percent of genes the most highly expressed in the transcriptome. Data of H3K27me3-marked genes in Arabidopsis roots are from^6^, expression data are from^31^, CLF-regulated genes are from^10^.

Our results demonstrate an original role for PRC2 and H3K27me3 in the modulation of gene with very high expression. In order to explore the extent of this original function for PRC2, we compared the profile of highly expressed genes in the *Arabidopsis* root transcriptome^31^ with the genome-wide distribution of H3K27me3 in the roots^6^. We used in this case the transcriptome of plants grown under N-rich condition in order to be consistent with the conditions used to perform the epigenomic map. Among the most highly expressed genes in the transcriptome, we observed that 139 genes are targeted by H3K27me3 (Fig. 5, Table S2). This amount is obviously much lower than for poorly expressed genes, but it suggests that the regulation identified on *NRT2.1* may affect a substantial number of genes. We also analyzed whether highly expressed genes marked by H3K27me3 could be down-regulated by CLF, as we observed for *NRT2.1*. We therefore compared the list of highly expressed genes marked by H3K27me3 with the genes regulated by CLF in *Arabidopsis* roots^10^. 9 of the 139 highly expressed genes marked by H3K27me3 were found to be regulated by CLF (Fig. 5, Table S2), which is a similar proportion to that of poorly expressed genes (61 genes regulated by CLF on 803 genes with very low expression in the roots and marked by H3K27me3). Most compelling is the finding that the functional categories of hyperactive genes targeted by H3K27me3 in *Arabidopsis* roots are different from those of low expression genes marked by H3K27me3 (Table S3). Indeed, the set of low expression genes targeted by H3K27me3 is principally enriched in genes involved in the regulation of development, transcription and gene expression, as previously described^5,6,11^. In contrast, the list of very highly expressed genes targeted by H3K27me3 shows a significant enrichment in genes involved in metabolic processes and response to diverse stimuli (Table S3). In particular, it included gene categories related to nitrate transport and assimilation, as well as several other processes linked to mineral nutrition and secondary metabolism (Table S3). Such observation lends support to the conclusion that this original regulation by PRC2 in plants could mostly affect genes that are relevant to plant physiology and to response to the environment, including those linked to an essential function like mineral nutrition.

## Discussion

NRT2.1 is a major root NO_3_^−^ transporter and is essential for plant growth under limiting NO_3_^−^ availability^23–25^. The molecular mechanisms that control the level of *NRT2.1* expression are therefore crucial for plant growth and development. We show here that *NRT2.1* expression, which is one of the highest in the transcriptome, is modulated by PRC2, a chromatin-based regulator of gene expression known to be associated with strong gene repression. Examination of *NRT2.1* chromatin state reveals that this locus is targeted by H3K27me3 under both repressive and inductive conditions for gene expression, mainly directed by CLF. Decrease in H3K27me3 levels did not lead to induction of *NRT2.1* expression under repressive conditions. These observations lend support to the conclusion that H3K27me3 enrichment is not the main determinant of *NRT2.1* repression under N-rich condition, and are in agreement with the global view that loss of H3K27me3 alone is generally not sufficient to lead to a gain of expression^21^. Indeed, it is possible that induction factors specific to low N conditions are also required to activate *NRT2.1* in the absence of repressive chromatin marks. Unexpectedly, in the context of very high expression, loss of H3K27me3 leads to further increase in *NRT2.1* expression. This reveals a greatly unusual targeting by H3K27me3 to such a very highly expressed gene. Further increase in *NRT2.1* expression is observed in *NRT2.1*-expressing tissues, and coincides with an absence of misregulation of known regulators of *NRT2.1* expression. However, it should be noted that we cannot entirely rule out indirect effect of PRC2 loss-of-function. Notably, cell type-specific chromatin analysis in cortex and/or epidermis cells will be required to further explore the role of PRC2 in the regulation of *NRT2.1* high expression. We also observed that chromatin marks associated with active transcription, which would have been expected to be strongly present at *NRT2.1*, were surprisingly low at this locus. A strong enrichment of H3K27me3 at *NRT2.1* could explain such observation, at least for H3K36me3, which has been shown to be mutually exclusive with H3K27me3^33^. However, a dilution of *NRT2.1*-expressing cells in chromatin analysis performed with whole roots may also explain the lower than expected enrichment in chromatin marks associated with active transcription at the *NRT2.1* locus. Nevertheless, our results reveal an unusual chromatin state with high level of H3K27me3 and H3K4me3, and an original function for PRC2 in the regulation of the target gene.

Our results reveal that *NRT2.1* promoter under highly active conditions for expression is sufficient to drive H3K27me3 targeting to downstream sequences, and show that the effect of *clf* mutation occurs mainly at the level of promoter activity. Moreover, the induction of expression following loss of H3K27me3 varies substantially between *NRT2.1* and reporters of *NRT2.1* promoter activity. Indeed, induction of *ProNRT2.1:GUS* expression was clearly higher than the one observed for *NRT2.1* itself. This might translate additional mechanisms of transcriptional or post-transcriptional modulation targeted specifically to *NRT2.1* gene body or to *NRT2.1* mRNA. In addition, although regulation by CLF seems clearly directed to the *NRT2.1* promoter region, a larger genomic context at the *NRT2.1* locus is certainly also important for the regulation of its expression, and for the regulation by chromatin complexes. It is for instance interesting to observe that, although under repressive conditions, local chromatin interactions have been identified at the *NRT2.1* locus^34^, suggesting that chromatin conformation may have an influence on the expression of *NRT2.1*. The presence of multiple mechanisms to regulate *NRT2.1* is consistent, as this gene is essential for plants to survive in the majority of soil environments^27^.

Our analysis of previously published datasets reveals that a considerable number of genes showing very high expression also display H3K27me3 targeting. Although we cannot rule out that this overlap could be due to a combination of different cell types in which genes are either highly expressed or marked by H3K27me3, it supports the possibility that our observations made on the regulation of *NRT2.1* apply to many other genes. Interestingly, genes that would be targeted by this regulation fall into specific functional categories. Most of them are directly involved in metabolic processes, including mineral nutrition. These genes may be representative of fundamental mechanisms, for which a balance between high expression and gene integrity would be essential for plant physiology. In conclusion, our work provides the first example of a totally unexpected function of PRC2 in *Arabidopsis* in the modulation of one of the most highly expressed gene in the transcriptome, in a context of very strong transcription. This study opens a new route for further investigation concerning the role of PRC2 in the control of the expression of highly transcribed genes.

## Material and Methods

### Plant material and growth conditions

The *Arabidopsis thaliana* accession used in this study was *Col-0*. Mutant alleles and transgenic plants used in this study are *clf-29*^35^, *swn-3*^4^, *ProNRT2.1:GUS*^29^, *ProNRT2.1:LUC*^28^, *ProCLF:CFP:CLF;clf-29*^17^. Most of experiments were performed using roots from 7 days-old seedlings grown under a long-day photoperiod (16 h light and 8 h dark) on vertical MS/2 plates without nitrogen (*PlantMedia*) supplied with 0.8% agar, 0.1% of sucrose, 0.5 g/L MES and the appropriate concentration of nitrogen as described in figure legends.

### RNA extraction and expression analysis

Root samples were frozen in liquid nitrogen and total RNA was extracted using TRI REAGENT (*MRC*), DNase treated (RQ1 *Promega*), and reverse transcription was achieved with M-MLV reverse transcriptase (RNase H minus, Point Mutant, *Promega*) using an anchored oligo(dT)_20_ primer. Accumulation of transcripts was measured by qRT-PCR (LightCycler 480, *Roche Diagnostics*) using the SYBR^R^ Premix Ex Taq^TM^ (*TaKaRa*). Gene expression was normalized using *ACT2* as an internal standard. Sequences of primers used in qPCR for gene expression analysis are listed in Supplementary File 1.

### ChIP experiments

ChIP experiments were performed as previously described^36^ with minor modifications. Nuclei were isolated using Nuclei Isolation Buffer (20 mM PIPES-KOH pH 7.6, 1 M hexylene glycol, 10 mM MgCl2, 0.1 mM EGTA, 15 mM NaCl, 60 mM KCl, 0.5% Triton X100, 5 mM beta-mercaptoethanol, protease inhibitor cocktail (complete tablets EASYpack, *Roche*)) and then resuspended in Nuclei Lysis Buffer. Chromatin was precipitated with 2.5 μg of antibodies against H3 (*Abcam* 1791), H3K27me3 (Millipore *07-449*), H3K4me3 (Diagenode *C15410030*), H3K36me3 (Abcam 9050), H3K9ac (Agrisera *AS163198*). Immunoprecipitation of CFP::CLF was performed using GFP-Trap MA (*Chromotek*). Immunoprecipitated DNA was purified with IPURE Kit (*Diagenode*) and resulting DNA was analyzed by qPCR analysis. ChIP experiments were normalized using H3 level as an internal standard. Data are presented as the percentage of H3K27me3 enrichment over H3 enrichment, using the following formula: 2^−(Cp IP H3K27me3 – Cp IP H3)^ × 100. For CFP::CLF immunoprecipitation, experiments were normalized using an INPUT (10% of sample adjusted to 100%). Data are presented as the percentage of CFP:CLF enrichment over input, using the following formula: (2^−(Cp IP H3K27me3 – Cp Input)^ × 100)/10. Sequences of primers used in qPCR for ChIP experiments are listed in Supplementary File 1.

### GUS histochemical staining and *Arabidopsis* root cross-section

Plants were harvested and prefixed 45 minutes at room temperature in 50 mM NaPO_4_ pH 7, 1.5% formaldehyde, 0.05% Triton X100. Plants were washed 3 times in 50 mM NaPO_4_ pH 7 before staining in 50 mM NaPO_4_ pH 7, 0.5 mM ferricyanide, 0.5 mM ferrocyanide, 0.05% Triton X100, 1 mM X-Gluc 30 minutes under vacuum following by 2 h incubation at 37 °C. Three other washes in 50 mM NaPO_4_ pH 7 are performed before another fixation under vacuum for 15 minutes in 2% paraformaldehyde, 0.5% glutaraldehyde, 100 mM NaPO_4_ pH 7 following by 24 h incubation at 4 °C. Samples were cut into 1 cm fragments and mature parts of roots were subjected to gradual dehydration to overnight incubation in 100% EtOH. Inclusions were performed using Technovit 7100 cold-curing resin *(Heraeus Kulzer* performed according manufacturer’s recommendations). Transversal sections of 5 µm were realized using a microtome (*Leica* RM2165) and observed in water under BH2 microscope with color view soft imaging system (camera) and Cell^A software.

### Gene Ontology analysis

Gene ontology has been analyzed using BINGO under Cytoscape environment, using Biological Process file, and a significance level of 0.05.

### Data analysis and presentation

Mean ± SE is shown for all numerical values, and based on at least 3 biological replicates. Statistical significance was computed using a two-tailed Student’s t-test. Significance cutoff: *p < 0.05, **p < 0.01, ***p < 0.001.

## Electronic supplementary material

Supporting Information

Table S1

Table S2

Table S3
