# Supplementary material for: Polycomb Repressive Complex 2 attenuates the very high expression of the Arabidopsis gene NRT2.1
Source: Sci Rep. 2018 May 21;8:7905. doi: 10.1038/s41598-018-26349-w (PMC5962593; doi:10.1038/s41598-018-26349-w)
Supplement: Supplementary file 1 — Supporting Information [file 41598_2018_26349_MOESM1_ESM.pdf]

Supplementary Information

Polycomb Repressive Complex 2 attenuates the very high expression of the *Arabidopsis* gene *NRT2.1*

Fanny Bellegarde[1], Léo Herbert[1], David Séré[1], Erwann Caillieux[2], Jossia Boucherez[1], Cécile Fizames[1], François Roudier[2,3], Alain Gojon[1], Antoine Martin[1*]

[1] BPMP, CNRS, INRA, SupAgro, Univ. Montpellier, Montpellier, France.

[2] Institut de Biologie de l'Ecole Normale Supérieure, CNRS UMR8197, INSERM U1024, ENS, 46 rue d'Ulm, 75005 Paris, France.

[3] Laboratoire Reproduction et Développement des Plantes, Univ Lyon, ENS de Lyon, UCB Lyon 1, CNRS, INRA, F-69342, Lyon, France.

*For correspondence: antoine.martin@supagro.fr

Supporting Information Legends

Figure S1: Position of primers used for ChIP experiments. Schematic representation of target and control genes used in this study, and position of primers used for ChIP experiments.

Figure S2: Genome Browser view of H3K27me3 enrichment at the *NRT2.1* locus. Snapshot of H3K27me3 enrichment at the *NRT2.1* locus, obtained from the Epigara GBrowser (http://epigara.biologie.ens.fr/).

Figure S3: Expression levels of *ProNRT2.1:LUC* reporter line under limiting nitrate (0.3 mM NO_3^-) condition. Relative expression of *ProNRT2.1:LUC* by qRT-PCR in roots of 7-days old *WT* and *clf-29* plants grown under low nitrate (0.3 mM NO_3^-) condition. Quantification by qRT-PCR is shown as the percentage of *ACT2* transcript levels. Error bars represent standard errors of the mean based on 3 biological replicates.

Figure S4: CLF is associated to the *NRT2.1* locus under limiting NO_3^- condition. ChIP experiments were performed with chromatin from roots of 7-days old c*lf-29* plants carrying a *ProCLF:CFP:CLF* transgene, grown under low nitrate (0.3 mM NO_3^-) condition. Signals were detected by qRT-PCR and normalized against the input for *NRT2.1*, *LEC2* (positive control) and *ACT7* (negative control).

Figure S5: Expression of *NRT2.1* transcriptional regulators is not affected in *clf-29*. Relative expression of the main transcriptional regulators of *NRT2.1*. qRT-PCR in roots of 7-days old *WT* or *clf-29* plants grown under low nitrate (0.3 mM NO_3^-) condition. Error bars represent standard errors of the mean based on 3 biological replicates.

Supplementary file 1: Primers used in this study

Table S1: Expression values from transcriptomic experiments using Arabidopsis roots grown under limiting nitrate.

Table S2: List of highly or poorly expressed genes marked by H3K27me3 and regulated by CLF in *Arabidopsis* roots.

Table S3: Gene ontology analysis of highly or poorly expressed genes marked by H3K27me3 in *Arabidopsis* roots.

Figure S1

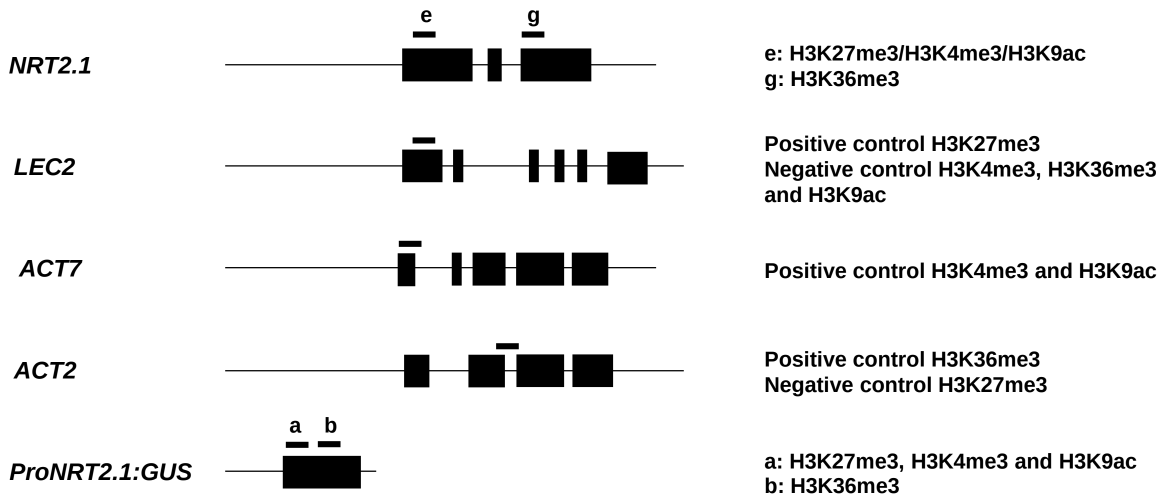

Figure S1

Figure S2

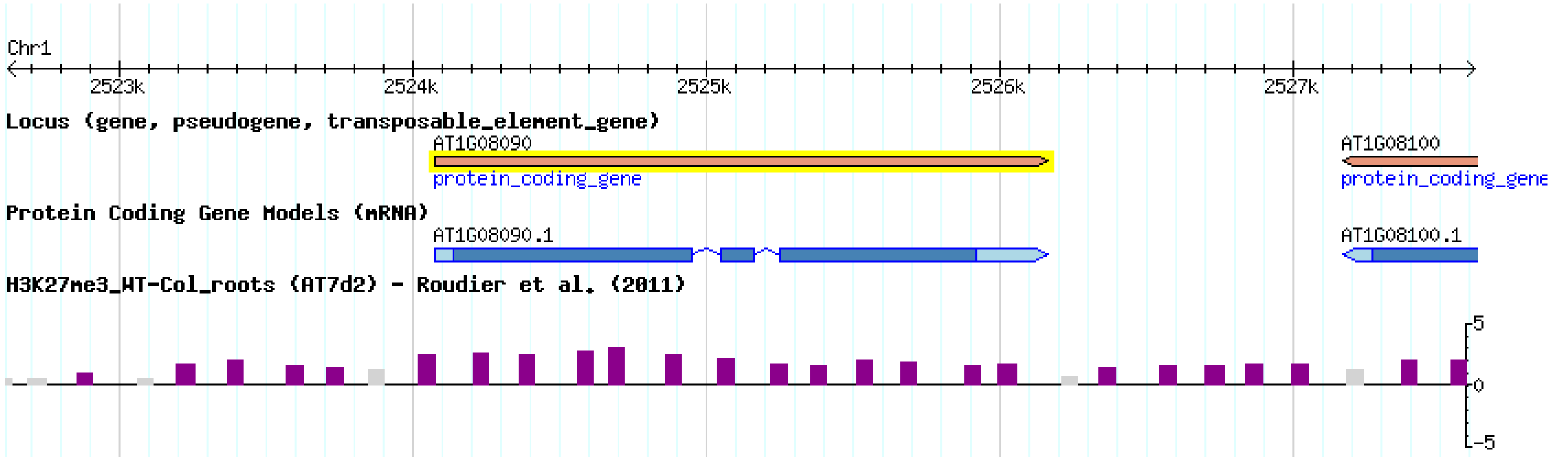

Figure S2

Figure S3

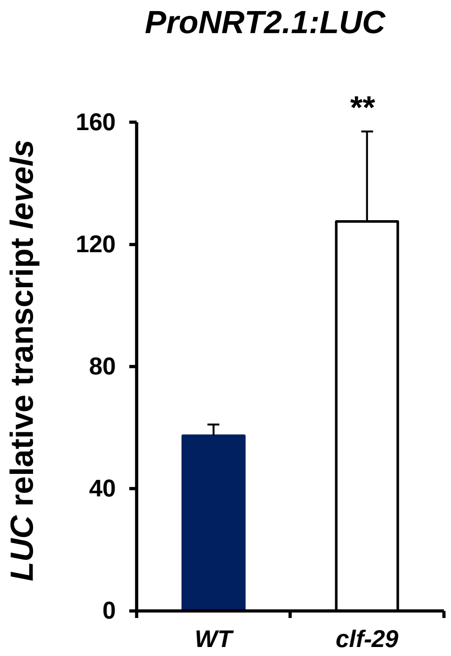

Figure S3

Figure S4

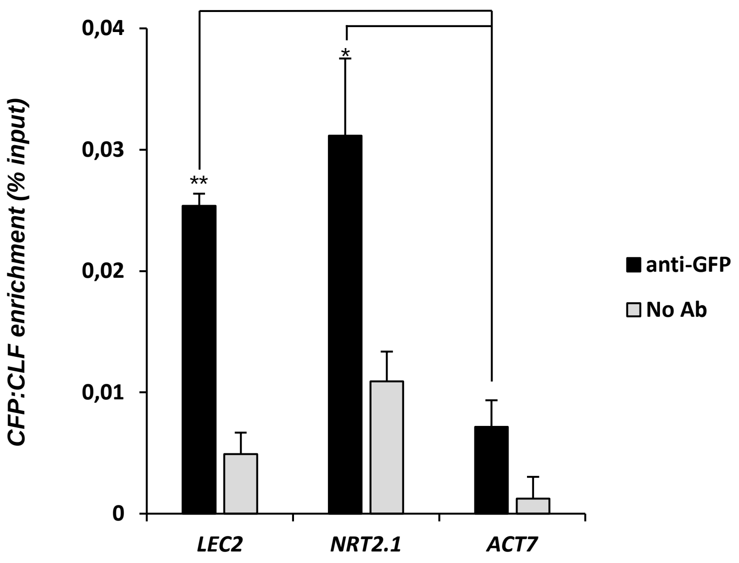

Figure S4

Figure S5

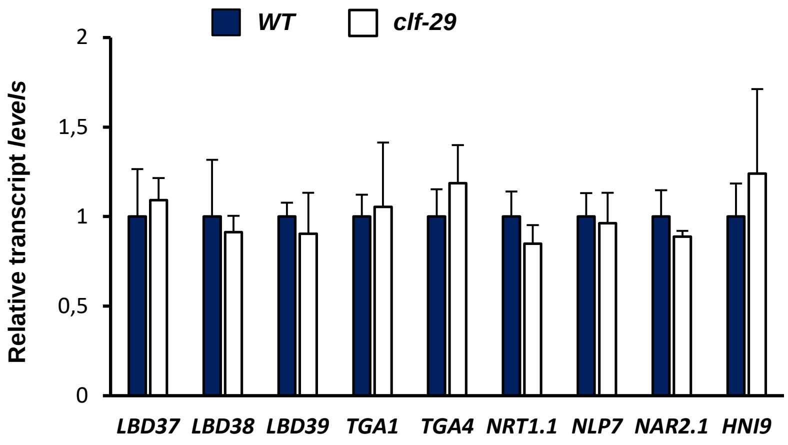

Figure S5

Supplementary file 1: Primers used in this study

Target gene	AGI	Forward/Reverse	Sequence	Application
LUC	-	R	GGTGTTGGAGCAAGATGGAT	RT- & ChIP-qPCR
		F	TCAAAGAGGCGAACTGTGTG	
GUS (a)	-	R	ACAGTTTTCGCGATCCAGAC	RT- & ChIP-qPCR
		F	TGGTCCGTCCTGTAGAAACC	
GUS (b)	-	R	TCGGCTTCAAATGGCGTATAG	ChIP-qPCR
		F	TTCGATGCGGTCACTCATTAC	
ProNRT2.1 (a)	*AT1G08090*	R	AGGATCTTCGGAACAGTGATG	ChIP-qPCR
		F	ACAAAGTGGTTCCTTCACGA	
ProNRT2.1 (b)	*AT1G08090*	R	TCGTGTGTGTACATGTCTTCATCT	ChIP-qPCR
		F	AGTTTGCCGTTTTTCTTTGTTCT	
ProNRT2.1 (c)	*AT1G08090*	R	CTACCCTTCCCTCATCTTGC	ChIP-qPCR
		F	TTTGGTTTCCACGCAATTTGACG	
ProNRT2.1 (d)	*AT1G08090*	R	GGGTTCCTAGCCAGTGTTGA	ChIP-qPCR
		F	TTTGGAAGGATATCGGCAAC	
NRT2.1 (e)	*AT1G08090*	R	TCGAACTTGGCCGTCTTGTC	ChIP-qPCR
		F	TGGGTGATTCTACTGGTGAGC	
NRT2.1 (f)	*AT1G08090*	R	GTTGAGATTCTCCCGGATGATAG	RT- & ChIP-qPCR
		F	CCATCTCTCGTGGATCTCTTTC	
NRT2.1 (g)	*AT1G08090*	R	ATGGAGTATCCGTAGAGAAGAACGA	ChIP-qPCR
		F	GTTGTAGATTCTGTGGTATGCCG	
NRT2.1 (h)	*AT1G08090*	R	CTGCTTCTCCTGCTCATTCC	ChIP-qPCR
		F	AACAAGGGCTAACGTGGATG	
LEC2	*AT1G28300*	R	TCATCACCGCCGCCATCTGC	RT- & ChIP-qPCR
		F	CGCTCGCACTTCACAACAGTCC	
ACT7	*AT5G09810*	R	AGCGAGAGATCGACAGAAGC	ChIP-qPCR
		F	GCATGCGTTGTGGTTTTATG	
ACT2	*AT3G18780*	R	CCCTCGTAGATTGGCACAGT	RT- & ChIP-qPCR
		F	GCCATCCAAGCTGTTCTCTC	
LBD37	*AT5G67420*	R	CAAAGCAGGACGTTGAGAATCC	RT-qPCR
		F	ATGGATTGAAACCGCCGATG	
LBD38	*AT3G49940*	R	ATGAGTTGCAATGGTTGTCG	RT-qPCR
		F	GGCCTTGAGCTTCAGGTGAT	
LBD39	*AT4G37540*	R	AGTTCCTGGTCCACAACATACC	RT-qPCR
		F	CTCCAACGTCCTGCTTTGTTTC	
NLP7	*AT4G24020*	R	CAGCTGCTGATGGAGAAGAGTAAG	RT-qPCR
		F	TTCTCCGACGGTGGAGGAAATG	
NAR2.1	*AT5G50200*	R	TGGAGCGTAGCATAGCTTAAC	RT-qPCR
		F	GGCAAGGATACGTTGAACATTAC	
TGA 1	*AT5G65210*	R	CTCTATTTATTCAGAGTAGTCCTCTGT	RT-qPCR
		F	GAACCAACGTAGGTTTGAGTTAT	
TGA4	*AT5G10030*	R	TATGTTGCAGTTTCTCCATACCT	RT-qPCR
		F	TCCTTTGACGGATCAACAACTT	
NRT1.1	*AT1G12110*	R	GCACATTGGCATTAGGCTTT	RT-qPCR
		F	CTCAATCCCCACCTCAGCTA	
HNI9	*AT1G32130*	F	GTGGCGAAAGGAAGTCAGAA	RT-qPCR
		R	CTCACCCTCTTCTGCCTGAG	
